# What Can Phages Tell Us about Host-Pathogen Coevolution?

**DOI:** 10.1155/2012/396165

**Published:** 2012-11-18

**Authors:** John J. Dennehy

**Affiliations:** ^1^Biology Department, Queens College, 65-30 Kissena Boulevard, Flushing, NY 11367, USA; ^2^The Graduate Center, The City University of New York, 365 Fifth Avenue, New York, NY 10016, USA

## Abstract

The outcomes of host-parasite interactions depend on the coevolutionary forces acting upon them, but because every host-parasite relation is enmeshed in a web of biotic and abiotic interactions across a heterogeneous landscape, host-parasite coevolution has proven difficult to study. Simple laboratory phage-bacteria microcosms can ameliorate this difficulty by allowing controlled, well-replicated experiments with a limited number of interactors. Genetic, population, and life history data obtained from these studies permit a closer examination of the fundamental correlates of host-parasite coevolution. In this paper, I describe the results of phage-bacteria coevolutionary studies and their implications for the study of host-parasite coevolution. Recent experimental studies have confirmed phage-host coevolutionary dynamics in the laboratory and have shown that coevolution can increase parasite virulence, specialization, adaptation, and diversity. Genetically, coevolution frequently proceeds in a manner best described by the Gene for Gene model, typified by arms race dynamics, but certain contexts can result in Red Queen dynamics according to the Matching Alleles model. Although some features appear to apply only to phage-bacteria systems, other results are broadly generalizable and apply to all instances of antagonistic coevolution. With laboratory host-parasite coevolutionary studies, we can better understand the perplexing array of interactions that characterize organismal diversity in the wild.

## 1. Introduction

The story of life is a story of coevolution. Reciprocal relationships among replicators, whether competing, consuming, or cooperating, are a fundamental force driving organic diversification. Darwin clearly recognized as much. After observing *Angraecum sesquipedale* Thouars, a large Madagascan orchid with a foot-long nectary spur, he declared “in Madagascar there must be moths with proboscises capable of extension to a length of between ten and eleven inches!” [1–3]. Darwin made this connection because he realized that the long spur was a product of coevolution, or coadaptation as he called it, between flower and moth.

Orchids use nectar located at the base their spurs to entice pollinators to transfer pollen from one plant's anther to another's stigma so that fertilization can occur. However, pollinators with proboscises longer than an orchid's spur would not need to contact the flower to access its nectar and will remain free of pollen. Thus, a longer spur forces pollinators to dig deeper into the flower to drink its nectar, and more pollen adheres to the pollinator's body for transfer to the next orchid [4]. Therefore, long-spurred orchids likely reproduced more prolifically than did short-spurred orchids and came to characterize the species. By contrast, natural selection favors pollinators with longer proboscises because they are better able to drink the orchid's nectar [4] and are more likely to survive and reproduce. This natural tension between exploitation and reward has promoted repeated cycles of adaptation and counteradaptation, leading to greatly exaggerated spurs and proboscises.

Coevolution consists of reciprocal, adaptive genetic changes among two or more species [5]. In addition to morphological changes, coevolution may be important in many biological phenomena such as the evolution of sex [6, 7], virulence [8], drug resistance [9], immune defense [10], life histories [11], the maintenance of genetic diversity [12–14], speciation [15, 16], and community structuring [17, 18]. However, the theoretical and experimental analyses of coevolution did not begin in earnest until the 1950s and 60s (Thompson provides an excellent analysis of the historical underpinnings of coevolution studies [19]). Early study organisms included flax and flax rust [20–22]; butterflies and plants [23]; ants and acacias [24]. A common problem faced by biologists is that observing coevolutionary dynamics in field and laboratory studies of macroorganisms is difficult to achieve. As Ehrlich and Raven pondered in their influential paper, “without recourse to long-term experimentation on single systems, what can be learned about the coevolutionary responses of ecologically intimate organisms?” The subtext here is that, since evolution occurs over the course of many generations, long-term experimentation can be readily accomplished using short-lived organisms, such as microorganisms. 

Nonetheless, laboratory-based experimental coevolution of microorganisms did not gain favor until the 1970s and 1980s [25–33]. Here, host-parasite coevolution was examined in the context of interactions between bacteria and their bacteriophage parasites. Such studies have many advantages such as ease of control and replication, short generation times and rapid evolution, easy dissection of genetic changes associated with adaptation, and the ability to archive organisms for future study [34, 35]. 

In addition to providing important information regarding phage-host dynamics, these coevolution studies can contribute to our understanding of host-parasite dynamics among higher organisms. In principle, the fundamental laws of natural selection and adaptation are universal across all organisms. The factors governing coevolution among phage and their bacterial hosts should not be markedly different from that of a virus and a multicellular organism. Virus immune system evasion is conceptually similar to phage avoidance of host restriction or CRISPR systems [36, 37]. For example, simian immunodeficiency virus epitopes readily mutate to evade cytotoxic T-lymphocytes, occasionally via single-nucleotide substitutions [38, 39]. This situation is analogous to the mutation of a bacterial receptor to prevent phage binding or the mutation of a phage nucleotide sequence to prevent restriction. In this paper, I review recent contributions of laboratory experimental studies using bacteriophage to our broader understanding of host-parasite coevolution. I pose questions germane to coevolutionary theory, and then I discuss empirical evidence from phage studies that address these questions.

## 2. What Are the Mechanisms of Coevolution?

Coevolutionary theory covers a broad range of biological phenomena [40]; thus, it is not surprising that numerous models have been proposed [41]. Some, such as diffuse coevolutionary models, are not especially amenable for testing in laboratory microcosms in experimental evolution studies. Instead, I focus here on two fundamental aspects of coevolution: the underlying genetics of coevolution and the nature of the selective forces driving coevolutionary change. These facets of coevolutionary theory have been broadly tested using experimental populations of bacteria and phage. 

**Figure 1:**
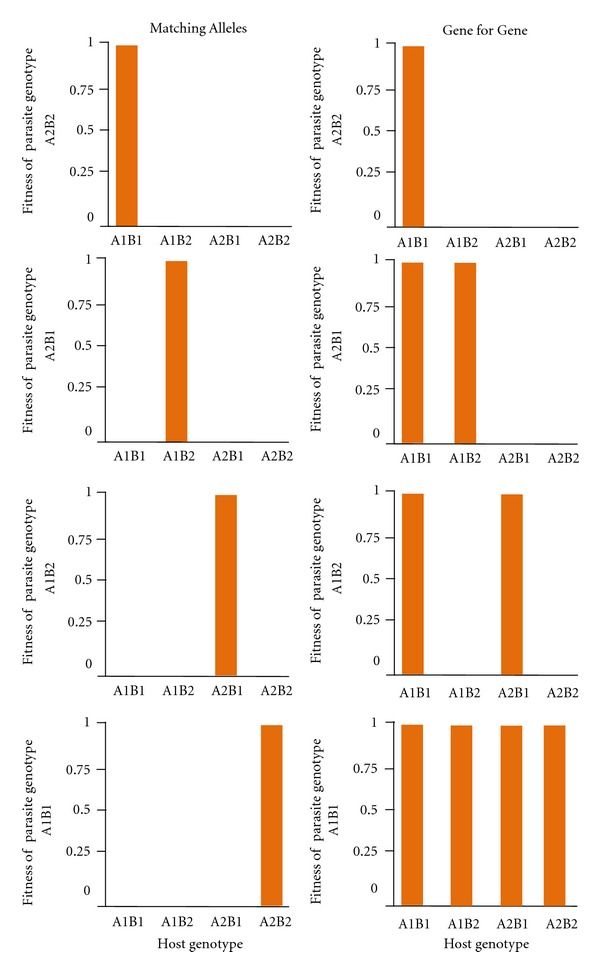
Fitnesses are shown for four parasite genotypes on each host genotype as implied by MA (left) and GFG (right) coevolutionary models. In the MA model, a parasite's genotype must precisely match a host's genotype in order to avoid recognition by the host's immune system and reproduce in the host. One consequence is that the number of parasite alleles matches the number of host alleles. By contrast, in the GFG model, a host is susceptible to all parasites except those for which it has a corresponding resistance allele. In this scenario, parasite alleles can outnumber host alleles. Figure modified from [42, 44].

**Figure 2:**
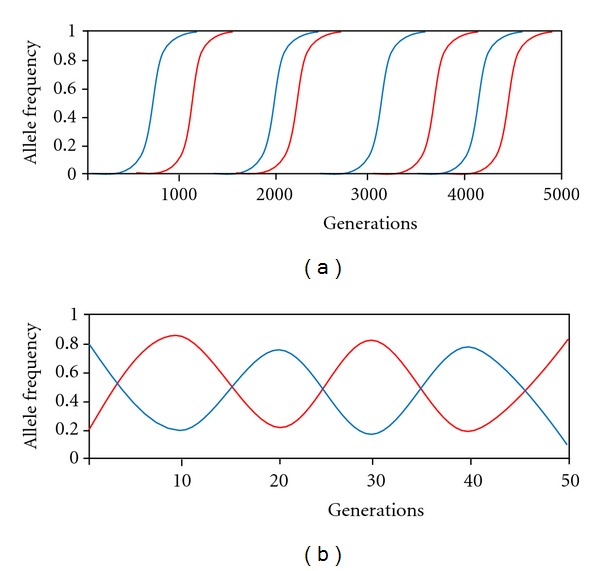
Plots represent allele frequency changes over time as predicted by GFG (a) and MA (b) models of coevolution. In the GFG model, directional selection fixes host (blue) and parasite (red) alleles arising via mutation. Each specific host resistance allele interacts with a specific parasite avirulence gene. Parasites counter host resistance via mutations in avirulence genes. Over time, genetic changes accumulate in both populations. By contrast, virulence and resistance alleles persist as dynamic polymorphisms in the MA models. Here parasites (red) become specialized for a common host genotype (blue), reducing its fitness. Over time, this host genotype will decline in frequency as less common genotypes are favored because of reduced parasite load. Reduced host frequency reduces the benefits of parasite specialization on this host relative to more common host genotypes. Reduced parasite load leads to increased host fitness, causing the cycle to repeat. Figure modified from Woolhouse et al., 2002 [5].

With regard to the genetics of coevolution, two simple models have proven popular: Flor's Gene for Gene (GFG) model [20] and the Matching Alleles (MA) model [42]. Kerr describes the GFG model as specifying that “for each [product] determining resistance in the host there is a corresponding [product] for avirulence in the parasite with which it specifically interacts” [43]. In other words, hosts are resistant if they have alleles allowing for the recognition of a specific avirulence allele presented by the parasite. Parasite alleles not specifically countered by a host allele allow a parasite to infect a host. One of the GFG model's key features is the existence of specialist and generalist parasite genotypes. Indeed universally virulent parasites are possible, and consequently there can be more parasite genotypes than host genotypes (Figure 1). However, fitness costs offset the benefits conveyed by broader virulence; thus, universally virulent genotypes may be selected against. Furthermore, the GFG model implies the replacement of virulence and resistance alleles by directional selection, leading to increased population differentiation over time (Figure 2). By contrast, in the MA model, parasite genotypes must exactly match host genotypes in order to evade recognition by the host's immune system and reproduce in the host. In this sense, MA models imply a self/nonself-recognition system where the host is unable to recognize successful parasites as foreign. Hence, a consequence of the MA model is that there should be the same number of parasite genotypes as host genotypes (Figure 1). The MA model is usually characterized by frequency-dependent selection on virulence and resistance alleles; thus, allele frequencies, but not alleles, change over time (Figure 2).

The MA and GFG models make contrasting predictions. For example, the MA model implies that the costs of resistance or infectivity are similar among all alleles, whereas costs can vary in GFG models [45, 46]. Moreover, the MA model predicts local adaptation and specialization, while the GFG model does not [47–49]. Generally the MA model is expected to result in frequency-dependent selection as opposed to GFG's arms race dynamics. These latter two criteria may not be absolute; thus, they may not be robust differentiating predictions [14, 42, 50, 51]. Agrawal and Lively point out that GFG and MA are likely endpoints in a continuum of parasite specificity [42]. In the wild, coevolutionary systems may show characteristics of both models.

Both models are attractive because of their relative simplicity, but may only be applicable to a relatively narrow range of organismal interactions. For example, they cannot account for active parasite choice in determining the host to infect [41]. In addition, it is likely that many coevolving traits are continuously varying traits controlled by multiple loci, which are more challenging to model genetically. However, simple systems such as phages and viruses might be precisely the sort of places one would expect relatively uncomplicated genetics. 

The GFG and MA models have been explored experimentally in phage-host systems, and the available evidence mostly supports the GFG model of antagonistic coevolution [47, 52–57]. For example, Brockhurst et al. report that generalist pathogens arose in well-mixed communities of *Pseudomonas fluorescens* and the phage Φ2 [52]. As GFG coevolution is predicted to result in selection for generalist genotypes in large, panmictic populations, these results matched expectations. These results were supported by studies by Morgan et al. and Scanlan et al., who found an increasingly wide range of host and parasite genotypes showing considerable variation in specificity [47, 54, 56]. Furthermore, in a meta-analysis of 37 experimental studies, Flores et al. found that host-phage infection networks show a nested pattern [58], which is a predicted outcome of GFG coevolution [42]. However, aspects of some studies better matched the MA model. For example, parasites were found to be locally maladapted, implying MA-like dynamics [46, 54, 59].

Overall, the balance of support seems to lie within the GFG model, but further experimental investigations using conditions designed to specifically differentiate the two are warranted. One consideration is that resource abundance may lower the costs of resistance (or infectivity) and lead to increased directional selection characteristic of arms race dynamics [60]. The assumption here is that, in resource-rich environments, the progressive accumulation of genetic changes characteristic generalist species is less likely to be disfavored by natural selection [48]. By contrast, where costs are present, fitness can be negatively frequency-dependent, leading to fluctuating selection.

## 3. Do Hosts and Parasites Experience Arms Races?

Many treatments assume that host-parasite relationships are characterized by evolutionary arms races; however, precisely defining “arms race” is difficult because the nature of the selective forces acting on coevolving populations are occasionally conflated [5]. Woolhouse et al. differentiate Red Queen dynamics from arms race dynamics [5]. In the Red Queen scenario (usually associated with MA models), frequency-dependent selection favors rare genotypes that are resistant to or infective of their antagonists (Figure 2). These populations are expected to be genetically diverse as resistance and infectivity polymorphisms are maintained over time [5, 14]. By contrast, arms race dynamics (usually associated with GFG models [44, 61, 62]) entail the replacement of one genotype with another due to selective sweeps, resulting in continual improvements in both populations over time (Figure 2). 

 Actually demonstrating coevolution, let alone arms races, in bacteria-phage systems has proven elusive. For example, many studies have reported that bacteria and phage achieve a stable state in continuous culture (See Table 1) [25–28, 30, 31, 33, 63–67]. However, in these studies, bacteria tended to stabilize at a density similar to that of controls, and phages persisted at a relatively low density. It is difficult to argue that this coevolution. A more likely explanation for these results is that while most bacteria have achieved resistance to the phages, some sensitive bacteria have found refuge in biofilms that form along the chemostat's walls. Presumably, this sensitive bacterial population is able to support a low-density phage population. Indeed, many of the cited studies found considerable levels of susceptible bacteria even after resistant bacteria came to dominate the culture. This hypothesis is supported by Schrag and Mittler's finding that abolishing biofilm formation by serial transfer resulted in phage extinction [68].

Alternatively, sensitive strains may be protected by a “numerical refuge” when they are rare. The continued presence of sensitive strains of hosts, and their parasites, may be simply a consequence of the cost of resistance and the frequency of parasites [27, 28, 32, 65, 68]. When sensitive strain densities are low, phage densities will also decline. When phage densities are low, sensitive strains will outcompete resistant strains by virtue of their greater growth rates; thus, they will again increase in number. Phage densities rebound until sensitive hosts are again depleted. Thus, sensitive strains, and their parasites, are maintained in populations through time. Although Schrag and Mittler did not find evidence supporting this hypothesis [68], the main message here is that the mere presence of phage is not evidence of coevolution unless it can be demonstrated that the phages are directly responding to host resistance. If only the hosts are responding, it is merely evolution, not coevolution.

 Assuming that phages would go extinct in the absence of sensitive hosts, it would appear that host victory can be a frequent occurrence in bacteria-phage coevolution experiments. A common explanation for the frequency of host victory is that bacterial hosts have more evolutionary potential than their phage parasites [30, 73]. Bacteria can evolve phage resistance by loss or alteration of phage receptor, but phage must gain the ability to bind to and productively infect a resistant host. A fair assumption is that the former types of mutations outnumber the latter. Given their high mutation rates and large population sizes, bacteria are likely able to search the phage-resistance sequence space rapidly. Larger genomes may provide bacteria greater evolutionary potential as they have a more flexible repertoire of targets able to be mutated to provide resistance [32, 74]. In addition, an important consideration is the bacteriophage population size following the inherent delay between a bacterial population's crash and eventual recovery. Phage populations will degrade quickly over time unless replenished by phage reproduction. Once phage populations are knocked down by host resistance, their slightly higher mutation rates are probably insufficient to compensate for the smaller frequency of host range mutations. In fact, Lenski and Levin estimated that, given a 15 mL chemostat containing bacteriophage at an equilibrium density of 1 × 10^6^/mL and a population-wide mutation rate of 1.6 × 10^−5^ per hr, it would take more than 7 years for a phage host range mutant to appear [30].

 Despite the frequency of host victory, some phage and bacteria antagonists have been shown to undergo multiple rounds of coevolution, leading to a state of mutual persistence (Table 1) [29, 31, 60, 66, 70–72]. For example, Marston et al. observed at least 4 cycles of adaptation/counteradaptation between the cyanobacterium *Synechococcus* and the phage RIM8 during a 6-month chemostat experiment [72]. Between 4 and 13 distinct phage host range phenotypes and between 4 and 11 newly evolved *Synechococcus* phage resistance phenotypes were identified. This study demonstrates that there is no fundamental constraint on the ability of phage to coevolve with their hosts. 

 Given that multiple rounds of host-phage coevolution are possible, is the coevolution best characterized by arms race or Red Queen dynamics? Buckling and Rainey reported that coevolution between *P. fluorescens* and phage Φ2 was directional, with hosts and parasites becoming resistant to or infective of a wider range of antagonists over time [60]. Subsequent studies using the same system found similar results [56, 57, 75, 76]. However, Hall et al. found that coevolutionary dynamics transitioned from arms race-like to Red Queen-like over the course of their experiments as resistance and infectivity measures leveled off at intermediate values [77, 78]. These results were attributed to the escalating costs of generalism, rather than lack of genetic potential for increased infectivity or resistance because universally infective phages and universally resistant hosts were found in some populations. The costs associated with generalism probably prevented host resistance and phage infectivity from increasing indefinitely. Interestingly a study using soil media instead of the standard laboratory agar reported Red Queen-like dynamics in soil, a lower productivity habitat, probably due to the increased costs of resistance and infectivity in this habitat [79]. 

## 4. Are Tradeoffs Associated with the Evolution of Resistance?

A common paradigm in evolutionary biology is that organismal phenotypes are constrained by limited resources. Evolution, then, tends to allocate resources such that fitness is maximized [80–82]. If resources are dedicated to pathogen resistance or if resources are acquired less efficiently due to a resistance phenotype, then fewer resources can be allocated towards reproduction. Thus, the evolution of resistance to a pathogen may entail a fitness cost in the host organism. Intuitively this makes sense since defense mechanisms such as biofilms, restriction enzymes, or receptor losses should incur metabolic costs. Indeed, the fitness cost of phage-resistance mutations arising during coevolution has been repeatedly demonstrated [28, 30, 60, 63, 64, 67, 71, 83–89]. Only a few studies failed to find resistance-associated fitness costs were assessed [30, 64, 66, 68, 70]. 

Since the mechanism of resistance to phage infection is often alteration or loss of a receptor [32, 90], resistance mutants may experience a reduction in metabolic efficiency or another important life characteristic. It is likely that resistance-associated fitness costs are ubiquitous, but not always detectable under laboratory experimental conditions. The resistance of *P. phaseolicola* to the phage Φ6 is a prime example. Φ6 infects *P. phaseolicola* by binding type IV pili. Nonpiliated mutant *P. phaseolicola* are resistant to Φ6 [91] and rapidly arise in coevolution experiments [70]. These nonpiliated mutants experience no fitness cost in the laboratory microcosm, but are likely poorly fit in the wild because *P. phaseolicola* use pili to attach to the leaves of plants [70, 92]. This finding suggests that cost-free phage-resistance observed in this study is likely an artifact of experimental conditions. We should expect that tradeoffs associated with resistance and infectivity will generally characterize host-parasite interactions. In the next section, I address host infectivity and the evolution of ecological specialization. 

## 5. Does Coevolution Lead to Ecological Specialization?

A common expectation is that host-parasite coevolution should lead to parasite ecological specialization [93–97], although this expectation tends to contradict the GFG model. Tradeoffs inherent to resource exploitation should favor genotypes that use resources more efficiently than competing genotypes. As the match between genotype and optimal phenotype is refined, parasites should be increasingly limited to specific host types. Indeed, bacteriophages tend to have restricted host ranges although some exceptions exist [98]. 

Since host range is a major concern of viral biology, particularly with respect to emerging infectious diseases, patterns observed among phages could be informative. Most phages reared on a single host will increase fitness on that host with concomitant reductions in fitness on other hosts [99–101]. Does specialization lead to evolutionary cul-de-sacs or can viruses easily reverse gears and broaden host range? Several studies have addressed this question and found, contrary to expectations, coevolution led to increased generalism among phages [55, 57, 60, 77, 78, 86]. Typically, in these studies, infectivity of a population of phages was assayed on bacterial clones isolated following each serial transfer [57, 60]. The presumed mechanism of increased generalism is reduced specificity of binding to host receptors [60] and is considered to be a general consequence of GFG coevolution. However, generalism did not increase without limit. In fact a cost of generalism among phages has been frequently demonstrated [28, 57, 78, 101, 102]. The cost of generalism was usually more pronounced in lower productivity habitats [55, 86, 103] (but see Forde et al. for a counterexample [104]), perhaps as a consequence of lower encounter rates, reduced mutation supply and increased host resistance [103], and the costs associated with resistance [60]. In addition, virus generalists may show reduced performance in novel environments [105]. To extrapolate to other host-parasite systems, we might expect that generalism may be more difficult to evolve in systems where population characteristics are reduced compared to phages (i.e., lower mutation rates, smaller population sizes, and lower encounter rates). Furthermore, other host types may not be as productive as bacterial hosts [106]. Lower-productivity habitats conform to the traditional definition specialization. 

## 6. Does Coevolution Increase Virulence?

 Since lytic phages do not conform to the traditional definition of virulence (i.e., parasite-imposed reduction in host fitness), virulence in lytic phages is commonly assessed through phage reproductive rate [107]. Declines in reproductive rate imply reduced virulence, and vice versa. What effect, then, does coevolution have on virulence? The data seem to suggest that antagonistic coevolution reduces phage virulence [28, 57, 71, 77] (but see Chen and Baric for a counterexample [108]). This phenomenon seems to be distinct from the reduction of virulence associated with selection for increased transmission (e.g., rabbit myxomatosis in Australia [109]) and is consistent with the ideas that poor environments [110] or host heterogeneities [111] should select for reduced virulence. Here, the cost of maintaining infectivity of a coevolved host is often a reduction in phage reproductive rate on the ancestral host. This cost may be a consequence of the pleiotropic costs of associated with infection of a new host variant [100, 112].

Other studies have shown that phage can increase the virulence to other hosts of pathogenic bacteria [113]. Here coevolution with the phage Φ2 led to the appearance of the *P. aeruginosa* mucoid phenotype containing the alginate virulence factor. Whether this is a specific artifact of the *P. aeruginosa* biology remains to be determined. 

## 7. Does Coevolution Accelerate Adaptation?

In a static environment, the rate of evolution tends to decrease over time as peaks on the adaptive landscape are approached [114]. One consequence of cyclic coevolution characteristic of Red Queen evolutionary dynamics is that the adaptive landscape and the selective forces acting on populations will frequently shift. We might expect that the rate of adaptation is increased in such coevolving populations relative to populations where one species is held in evolutionary stasis. Paterson et al. tested this hypothesis using the bacterium *P. fluorescens* SBW25 and its viral parasite, phage Φ2 [76]. In one treatment, ancestral bacteria from frozen stocks were added to purified phage isolated from serial transfer flasks. Thus, the bacterial genotype was held constant while the phage was allowed to adapt. In the other treatment, 1% of the phage/bacteria culture was transferred from flask to flask every 48 hours. Here, both interactors were allowed to coevolve. Paterson et al. found that the rate of molecular evolution was significantly higher in coevolving populations compared to the static control [76]. Similar results were obtained for the phage Q*β* and its host, *Escherichia coli* [71]. Other experiments demonstrated that population mixing [52] and resource availability [86] both significantly increased the rate of adaptation among coevolving phage. 

On the other hand, coevolution may limit responses to environmental change. Zhang and Buckling found that phage persistence in response to gradual temperature increases was greater for phage cultured with an evolutionarily constant host than for phage cultured with a coevolving host [115]. This reduction in fitness might result from a reduced population size and consequent reduced genetic diversity (Zhang and Buckling estimate 10-fold fewer beneficial mutations) or from the differential impact of temperature changes on coevolved phages [115]. These results imply that, although coevolution may accelerate molecular evolution, resulting organisms may be less resilient to ecological and environmental changes and less fit across a broad range of conditions. Much like two rival countries investing their resources in guns rather than butter, antagonistic coevolution may impose costs on populations. Hosts and parasites may be trapped into a devolutionary spiral because of reduced population sizes, increased costs of deleterious mutations [84], and increased spending on costly adaptations. This hypothesis is supported by studies documenting a cost of resistance for the host and a cost of infectivity for the parasite as described in the previous sections of this paper. On the other hand, coevolution may be a diversifying force that drives the differentiation of populations across heterogeneous landscapes [19, 116].

## 8. Does Coevolution Increase Organismal Diversity?

 A fundamental aim of evolutionary biology is to explain the diverse and “elaborately constructed forms” that populate the Earth around us [117]. The great variety of life cannot simply be a response to selection imposed by the abiotic environment; diversification must also be driven by what Darwin called, “mutual relations,” or interactions among organisms. Here, I describe experimental studies exploring the diversifying effects of antagonistic coevolution. For instance, Chao et al. reported that coevolving populations of phage and *E. coli* diversified into a complex community containing several different types of each organism [118]. However, this study also illustrates the difficulty of disentangling effects due to biotic and abiotic environments. As Schrag and Mittler later showed, bacterial diversification was largely driven by the spatial refuges provided by wall populations of bacteria [68]. In the absence of spatial structure, sensitive bacteria were replaced by resistant bacteria, with no net gain in organismal diversity. This result raises an important point. Diversification was driven by mutual relations, but only in the presence of habitat heterogeneity. As Thompson makes clear, populations respond to shifting patterns of interactions across space and time, the geographic mosaic [119]. Fragmentation of the natural landscape allows local populations of one species to adapt to local populations of other species in a genotype-by-genotype-by-environment interaction. As these interactions play out across larger spatial and temporal scales, and as different subpopulations interact or fail to interact, coevolutionary changes can result in speciation and increased biodiversity. 

Corresponding to this view of coevolution, Buckling and Rainey reported that sympatric diversity was reduced, but allopatric diversity was increased, among coevolving populations of the phage Φ2 and its host, *P. fluorescens* [53]. Usually *P. fluorescens* diversifies into several morphotypes when grown in spatially structured habitats (i.e., stationary flasks containing nutrient broth), due to the development of niche specialists [120]. However, when phages are added to the mix, populations were usually dominated by a single morphotype. Interestingly, when diversity was considered across replicate microcosms, global diversity was increased relative to phage-free controls. Antagonistic coevolution, then, apparently drove increased morphotype diversification by preventing the predictable sequence of morphotypes that appear in the absence of predation. Instead, resistance mutations arose in different genetic backgrounds among the various populations; thus, each population may have followed a unique evolutionary trajectory.

It should be noted that these results may not be broadly generalizable and may depend on biological details intrinsic to this system. Altering experimental conditions or changing phage-host pairings can lead to different patterns, namely, increases in sympatric diversity compared to controls [88, 89]. For example, Brockhurst et al. extended the findings of the Buckling and Rainey study by considering the effects of the removal of spatial structure (i.e., shaken flasks) [83]. Here, the lack of spatial structure prohibits the *P. fluorescens'* morphological diversification due to strong interspecific competition [120]. When spatial structure was removed, within and between population diversity increased relative to phage-free controls. The increased number of resistance morphs among the bacterial host populations may have resulted from the appearance of “mutators” or bacteria possessing increased mutation rates [121]. Thus, phages reduced diversity when spatial structure was imposed but increased diversity when spatial structure was removed. The addition of phages may allow the survival of weaker competitors in the experimental habitat [88]. Other experiments showed that host density, rate of parasite evolution [122], and resource availability [85, 123] all influenced host diversity. 

## 9. Summary

Experimental evolution studies employing phage and bacteria offer simple, replicable systems in which coevolutionary theory can be tested. Ease of sequencing makes tracking genetic changes over time practical and informative. Ability to freeze genotypes for later use allows time-staggered quantification of host resistance and parasite infectivity [60]. Easy modifications permit experimental analysis of the effects on coevolution of spatial structure [52, 124], community structure [125], resource availability [86, 126], gene flow [46, 47, 55, 59, 126–128], multiplicity of infection (ratio of phage to host) [75], mutation rate [129], and temperature [115]. 

Thus, with easy manipulation, populations can experience different widely variable coevolutionary conditions depending on environmental and ecological circumstances. Iterations varying coevolutionary circumstances have already produced much data. Studies have shown that coevolution can increase parasite specificity, virulence, adaptation rate, and diversity. 

Other salient findings highlight the importance of GFG coevolution, which gives rise to arms race population dynamics, but do not rule out allele frequency-dependence and Red Queen dynamics. Instead, both models probably operate with a high degree of context-dependency and may function at different times in the same population. Moreover, GFG and MA models may represent extremes along a continuum of pathogen specificity, and many coevolving genetic systems are expected to fall in between. Given that these experiments were performed in simple microcosms with a limited number of interactors, coevolutionary dynamics in the wild likely involves an even greater array of interactions and processes at all levels of the biological hierarchy. The complexity seen even in specific, as opposed to diffuse, coevolutionary systems poses a tremendous challenge, but much headway is to be made with laboratory experimental evolution studies. In light of the bewildering array of connections and influences that characterize wild populations, laboratory phage and bacteria models provide a tractable arena in which to better understand the causes and consequences of coevolutionary change.

## Figures and Tables

**Table 1:** Outcomes of experimental coevolutionary studies using bacteriophage and their bacterial hosts. In this table, the number of coevolutionary cycles and final states of laboratory experimental studies using bacteriophage are documented. Only those studies that did not explicitly manipulate experimental conditions (e.g., resource availability, gene flow) are presented; thus, this is not a comprehensive analysis of all bacteriophage coevolution studies.

Host	Parasite	Type	Duration	Cycles^a^	Outcome	Reference
*Escherichia coli *	T2	Chemostat	19 days	1-2?	Phage and host persistence	Paynter and Bungay, 1969 [69]
*E. coli *	T4	Chemostat	75 days	0.5	Host resistance^b^	Horne, 1970 [33]
*Plectonema boryanum *	LPP-1	Quasi-continuous	80 days	1-2?	Host resistance^b^	Cowlishaw and Mrsa, 1975 [25]
*P. boryanum *	LPP-1	Chemostat	90 days	2.5	Host resistance^b^	Cannon et al., 1976 [26]
*E. coli *	T7	Chemostat	68 days	1.5	Host resistance^b^	Chao et al., 1977 [28]
*P. boryanum *	LPP-DUN1	Chemostat	60 days	2.5?	Phage and host persistence, partial host resistance	Barnet et al., 1981 [29]
*Aphanothece stagnina *	Aph-1	Chemostat	60 days	2.5	Host resistance^b^	Barnet et al., 1981 [29]
*E. coli *	T2	Chemostat	12 days	1.5	Host resistance^b^	Lenski and Levin, 1985 [30]
*E. coli *	T4	Chemostat	21 days	0.5	Host resistance^b^	Lenski and Levin, 1985 [30]
*E. coli *	T5	Chemostat	12 days	0.5	Phage extinction	Lenski and Levin, 1985 [30]
*E. coli *	T7	Chemostat	12 days	1.5	Host resistance^b^	Lenski and Levin, 1985 [30]
*E. coli *	*λ*	Chemostat	135 days	1	Phage and host persistence, partial host resistance	Spanakis and Horne, 1987 [31]
*E. coli *	*λ*	Chemostat	12 days	0.5	Host resistance^b^	Schrag and Mittler, 1996 [68]
*E. coli *		Serial transfer	7 days	0.5	50% of lineages extinct after 7 days, all assumed extinct after 15 days	Schrag and Mittler, 1996 [68]
*E. coli *	T1X	Chemostat	12 days	0.5	Host resistance^b^	Schrag and Mittler, 1996 [68]
*E. coli *		Serial transfer	7 days	0.5	Phage extinction	Schrag and Mittler, 1996 [68]
*P. fluorescens *	Φ2	Serial transfer	100 days	Multiple	Phage and host persistence	Buckling and Rainey, 2002 [53]
*E. coli *	PP01	Chemostat	8 days	Multiple	Phage and host persistence	Mizoguchi et al., 2003 [66]
*Pseudomonas phaseolicola *	Φ6	Serial transfer	21 days	0.5	5/8 phage lines extinct, others persist on partially resistant hosts	Lythgoe and Chao, 2003 [70]
*Vibrio cholerae *	JSF4	Chemostat	15 days	0.5	Host resistance^b^	Wei et al., 2010 [64]
*V. cholerae *	B phage	Chemostat	6 days	0.5	Host resistance^b^	Wei et al., 2011 [63]
*V. cholerae *	T phage	Chemostat	25 days	0.5	Host resistance^b^	Wei et al., 2011 [63]
*E. coli *	Q*β*	Chemostat	54 days	2	Phage and host persistence	Kashiwagi and Yomo, 2011 [71]
*Synechococcus *sp.* WH7803 *	RIM8	Chemostat	167 days	>4	Phage and host persistence	Marston et al., 2012 [72]

^a^The evolution of host resistance to phage is considered a half cycle. A full cycle occurs when the appearance of resistant host is countered by a host range mutant.

^b^Continued persistence of sensitive bacteria in spatial refuges (i.e., wall population) allowed low-level phage persistence.
